# MiR-211 protects cerebral ischemia/reperfusion injury by inhibiting cell apoptosis

**DOI:** 10.1080/21655979.2020.1729322

**Published:** 2020-02-15

**Authors:** Wenyi Liu, Yuanqing Miao, Lin Zhang, Xiaolin Xu, Qi Luan

**Affiliations:** a Department of Anesthesia, The Affiliated Hospital of Qingdao University, Qingdao, Shandong, China; b Department of Medical Network Information Center, The Affiliated Hospital of Qingdao University, Qingdao, Shandong, China

**Keywords:** Ischemia/reperfusion, oxygen-glucose deprivation, apoptosis, Mir-211, P53-up-regulated modulator of apoptosis (PUMA)

## Abstract

MicroRNAs (miRNAs) have emerged as critical regulators of neuronal survival during cerebral ischemia/reperfusion injury. Accumulating evidence has shown that miR-211 plays a crucial role in regulating apoptosis and survival in various cell types. However, whether miR-211 is involved in regulating neuronal survival during cerebral ischemia/reperfusion injury remains unknown. In this study, we aimed to explore the biological role of miR-211 in regulating neuronal injury induced by oxygen-glucose deprivation/reoxygenation (OGD/R) and transient cerebral ischemia/reperfusion (I/R) injury *in vitro* and *in vivo*. We found that miR-211 expression was significantly downregulated in PC12 cells in response to OGD/R and in the penumbra of mouse in response to MCAO. Overexpression of miR-211 alleviated OGD/R-induced PC12 cell apoptosis, whereas miR-211 inhibition facilitated OGD/R-induced PC12 cell apoptosis *in vitro*. Moreover, overexpression of miR-211 reduced infarct volumes, neurologic score, and neuronal apoptosis in vivo, whereas miR-211 inhibition increased infarct volumes, neurologic score and neuronal apoptosis in vivo. Notably, our results identified P53-up-regulated modulator of apoptosis (PUMA) as a target gene of miR-211. Our findings suggested that miR-211 may protect against MCAO injury by targeting PUMA in rats, which paves a potential new way for the therapy of cerebral I/R injury.

## Graphical Abstract

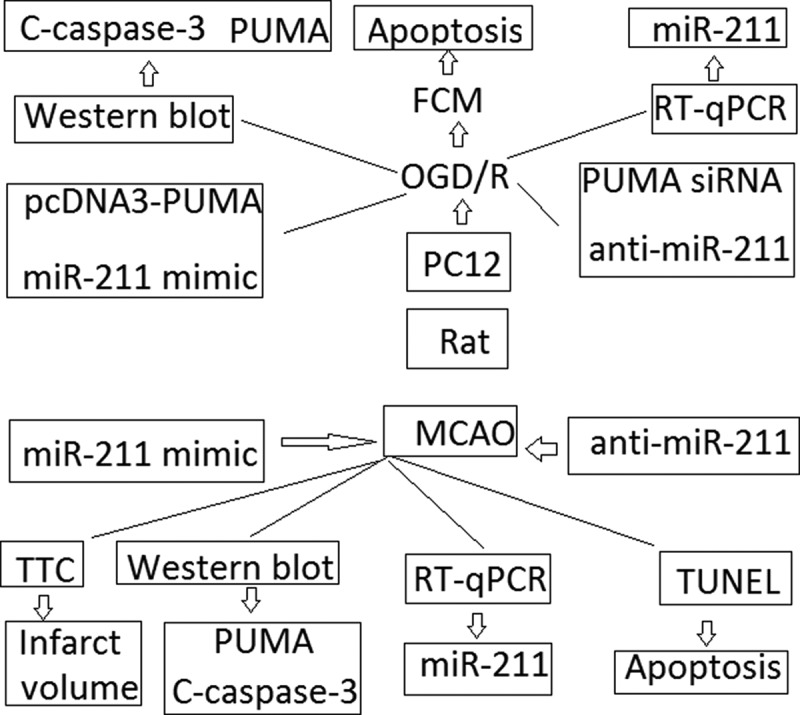

## Introduction

Ischemic stroke is a major cause of death and disability worldwide, and the clinical prognosis of acute cerebral ischemia is poor [1]. The early reperfusion after cerebral ischemia is essential for the viability and functional recovery of the brain; however, the arrival of blood oxygen to the ischemic tissue causes ischemia/reperfusion (I/R) injury, which induces further damage [2]. Currently, rarely pharmacological treatments can effectively ameliorate the sequelae from ischemic stroke. A promising and underway strategy to utilize thrombolytic therapies is tissue plasminogen activator (tPA) treatment [3]. However, the therapeutic time window of tPA has a restriction within 6 h. As a result, once happened, most stroke patients do not receive any specific pharmacology therapy, and only rehabilitative modalities would be expected to improve the functional outcomes [4,5]. Therefore, to develop the potential neuroprotective or therapeutic strategies to effectively improve the sequelae during stroke is imperative.

The development of new treatments requires a comprehensive understanding of the diverse mechanisms that are responsible for neuronal death during ischemic brain damage. Cerebral ischemia and reperfusion (I/R) can activate various programs of cell death, such as necrosis, apoptosis or autophagy-associated cell death [6–11]. Among these, apoptosis has been regarded as the key event for brain damage in cerebral ischemia [12]. Apoptotic cell death is induced by I/R, although the extent of cells dying via this modality is significantly lower than necrosis. Upregulation and activation of prodeath Bcl2 proteins (e.g., Bax, Bak, Bid, BNIP3, and Puma) and their translocation and integration into mitochondrial membranes occurs in ischemic cells [13–17]. Chen et al. [18] has recently reported that the expression of PUMA was significantly elevated in primary cerebral astrocytes during OSGD/R. Moreover, targeted down-regulation of PUMA by siRNA transfection significantly decreased the OSGD/R-induced apoptosis of primary cerebral astrocytes. In addition, OSGD and OSGD/R triggered the release of cytochrome c in astrocytes, indicating the dependence on a mitochondrial apoptotic pathway. Therefore, PUMA inhibition by reducing cell apoptosis may be ideal to protect ischemic tissue ischemia/reperfusion (I/R) injury.

MicroRNAs (miRs) are small endogenous RNA molecules (~21-25 nucleotides) that repress gene translation by hybridizing to 3~-UTRs of one or more mRNAs in a sequence-specific manner. By regulating expression of at least one-third of the human genome, miRs play a critical role in cell proliferation and differentiation, apoptosis, metabolism, and other biological processes [19]. MiRs have also been implicated in neurological diseases [20,21]. Others studies have demonstrated the essential role of miRs in the pathogenesis of ischemic injury in rodent stroke models, suggesting that miRs are potential therapeutic targets [22–24]. Wei et al [25]. reported that overexpression of the miR-29 family inhibited the OGD/R-induced elevation of ROS and reduction of MMP in HT-22 cells. In addition, administration of the miR-29 family suppressed proteins of Keap1, Bax and PUMA and increased Nrf2 expression. Ouyang et al. [26] reported that enforced miR-29a protects cell injury and mitochondrial function after ischemia-like stresses in vitro and delayed neuronal death after forebrain ischemia in vivo by targeting a pro-apoptotic BCL2 family member PUMA. It has recently found that targeting miR-711 protects neuronal cell and vascular from spinal cord injury by attenuating PUMA expression [27].These data indicated that there was a close relationship between PUMA and miRs expression in I/R induced cell injury.

The roles of miR-211 in tumor progression can be considered quite contradictory. For examble, miR-211 was upregulated in head and neck squamous cell carcinoma (HNSCC) and directly regulated TGFβ-RII to promote HNSCC progression and enhance c-Myc expression [28]. miR-211 is also a prosurvival microRNA that regulates chop expression in a PERK-dependent manner [29]. Additionally, miR-211 has been shown to function as a metabolic switch in melanoma cells by targeting the hypoxia inducible factor 1α (HIF-1α), and loss of miR-211–5p is expected to promote cancer hallmarks in human melanomas [30]. Recent studies have demonstrated that overexpression of miR-211 promoted colorectal cancer cell growth by downregulating the expression level of the CHD5 tumor suppressor *in vitro* and *in vivo* [31]. Recent studies reported that enforced miR-211 expression in the mesenchymal stem cells (MSCs) enhanced protection from myocardial I/R injury [32]. Another study reported that administration of miR-204/miR-211 mimics inhibited cell apoptosis and decreased the severity of the kidney injuries induced by Candidemia, as reflected by improved renal glomerular filtration rate, serum β2-microglobulin and blood urea nitrogen (BUN), and vice versa [33], suggesting that miR-211 overexpression could ameliorate I/R injury by inhibiting cell apoptosis.

In this study, we employed mouse PC12 neuronal cells upon *in vitro* ischemia oxygen-glucose deprivation/reoxygenation (OGD/R) model *in vitro* and rat cerebral ischemia/reperfusion (I/R) models *in vivo* to determine whether miR-211 is regulated by OGD/R or I/R challenge and, if yes, whether miR-211 takes part in the regulation of OGD/R or I/R injury and what is the underlying mechanism in the process of OGD/R or I/R injury. We found that the expression of miR-211 significantly decreased upon OGD/R or I/R challenge. Overexpression of miR-211 could protect the PC12 neural cells against OGD or I/R induced injury by targeting pro-apoptotic PUMA signal. The neuroprotective role of *miR-211* may be exploited for therapeutic intervention of I/R-induced neuronal injury.

### Materials and methods

#### Cell culture

Rat pheochromocytoma (PC12) cell line were obtained from American Type Culture Collection (ATCC, Shanghai, China) and passaged in Dulbecco’s modified Eagles’s medium (DMEM; Hyclone, USA) supplemented with 10% horse serum, 5% fetal bovine serum (Gibco, United States), penicillin (100 units/ml) and streptomycin (100 μg/ml) (Life technologies, NY). Cultures were maintained according to ATCC specified culture conditions at 37°C in a humidified incubator containing 95% air and 5% CO_2_. All treatments were performed on cells at 80% confluence.

#### Oxygen-glucose deprivation/reperfusion (OGD/R) model

In order to mimic ischemic-like conditions in vitro, PC12 cells were exposed to OGD/R injury as following: the culture media of the cell, DMEM, was replaced by Hanks Balanced Salt (HBSS; glucose concentration = 0 mg/dl) and then transferred to a hypoxic chamber (95% nitrogen and CO_2_ 5%) for 3 h. At the end of the OGD phase, the medium was replaced with growth medium containing 4.5 g/l glucose and cultured under normal conditions for 24 h reoxygenation [34]. Cells without OGD/R treatment were used as a control.

#### MiR-211 mimic or inhibitor transfection

miR-211 mimics (miR-211), miR-211 inhibitor (anti-miR-211), and negative control (miR-NC or anti-miR-NC) were purchased from Ambion (Austin, TX, USA). After seeded the PC12 cells at appropriated density, cells were transfected with miR-211 mimic or inhibitor transfection or negative control at a final concentration of 50 nm using Lipofectamine 2000 Reagent (Invitrogen, Carlsbad, USA) according to the manufacturer’s protocol. Cells were harvested at 24 h after transfection for the next study, then the cells above were subjective to OGD/R.

#### siRNA and plasmid transfection

PUMA inserts were cloned into the pcDNA3.1-Myc-His+ mammalian expression vector (Invitrogen/Life Technologies). Selected clones (pCDNA-PUMA) were analyzed for transgene expression levels and homogeneity of expression by immunoblotting. The PUMA siRNA oligos was purchased from Ambion Silencer Select oligos (Applied Biosystems): 5’-GCCUGUAAGAUACUGUAUAtt-3’. PC12 cells were transfected with 1 μg/ml DNA plasmid (pCDNA-PUMA) using Lipofectamine (Invitrogen). PC12 cells were transfected with PUMA siRNA with Lipofectamine RNAiMAX (Invitrogen) to a final concentration of 10 nM as the manufacture’s instruction.

#### Induction of MCAO

Male C57BL/6 mice were purchased from the Shanghai Institute of Animal. Focal cerebral ischemia was induced by intraluminal middle cerebral artery occlusion (MCAO) using a nylon monofilament suture. Briefly, mice were anesthetized with ketamine (100 mg/kg) and xylazine (10 mg/kg). After a midline skin incision, the left common carotid artery was exposed, and then its branches were electrocoagulated. A 2 cm length of 6–0 rounded tip nylon suture was gently advanced from the external carotid artery up to the internal carotid artery until regional cerebral blood flow (CBF) was reduced to <16% of baseline. After 60 min of proximal MCA occlusion, blood flow was restored by removing the suture. Changes in CBF at the surface of the cortex were recorded using a laser-Doppler flowmetry monitor (BPM2 System; Vasamedic). Sham control animals were subjected to similar operations to expose the carotid arteries without occlusion of the middle cerebral artery. After 60 min of MCAO, the mice were allowed to recover for 24 h. Arterial blood gases, mean arterial pressure, and heart rate were also monitored in selected animals 30 min before, during, and 30 min after MCAO. The rectal temperature was controlled at 37.0 ± 0.5°C during surgery with a feedback-regulated heating pad (Harvard Apparatus). After ischemic insult, mice were kept in an air-ventilated incubator at 24.0 ± 0.5°C. The animals were killed at 24 h of reperfusion, and the brains were quickly removed for infarct determination. All procedures using laboratory animals were approved by the Animal Care and Use Committee of the affiliated hospital of Qingdao University approved the animal experimental protocols, which strictly conforms to the NIH Guide for the Care and Use of Laboratory Animals (NIH Publication No. 85–23, revised 1996).

#### In vivo *experimental protocols*

In a second set of experiments, animals were randomly divided and pre-treated with either miR-211 mimic or miR-211 inhibitor or their control (each at final concentration of 30 pmol/g by tail vein) infusion 24 h prior to 1 h MCAO, and then sacrificed at 24 h of reperfusion for analysis of brain levels of miR-211, cell apoptosis and infarct determination.

#### 2, 3,5-Triphenyltetrazolium chloride (TTC) staining and infarct volume calculation

A neurological score was used to evaluate loss of neurobiological function according to the following 5-point rating scale: 0 = no deficit, 1 = failure to extend the left forepaw, 2 = decreased grip strength of left forepaw, 3 = circling to left by pulling the tail, 4 = spontaneous circling.

For measurement of infarct volume, the brains were sectioned into 4 coronal sections of 0.2–0.3 cm thickness. The sections were immersed in 2% 2,3,5-tripenyltetrazolium chloride (TTC) for 30 minutes at 37°C, then images were scanned into a computer and measured with imaging analysis software (Image J, NIH, USA). The presence or absence of infarction was determined by examining the TTC stain. The infarct volume (in cm3) for each section was equal to infarct area (in cm2) multiplied by the section thickness (0.2 or 0.3 cm). The total infarct volume for each brain was then calculated by summing up the infarct volume of all sections. To minimize the effect of edema on the accuracy of infarct volume measurement, the final infarct volume was corrected by a factor equal to the ratio of non-ischemic to ischemic hemisphere volumes.

#### Flow cytometric analysis

PC12 cell apoptosis was assayed by flow cytometry according to the protocol provided by the manufacturer after OGD. Briefly, the cells were washed twice with cold PBS before staining with FITC Annexin V and propidium iodide (PI) using the Annexin V-FITC Apoptosis Detection Kit I (BD Biosciences, Franklin Lakes, NJ, USA) for 15 min at room temperature in the dark. The stained cells were analyzed using flow cytometry within 1 h. The FITC Annexin V^+^/PI^−^ and FITC Annexin V^+^/PI^+^ cell populations were considered to represent necrotic and apoptotic cells.

#### Terminal deoxynucleotidyl-transferase-mediated dUTP nick end labeling (TUNEL) staining

TUNEL staining was applied to examine cell apoptosis in 10-μm frozen brain sections according to the manufacturer’s instructions (Roche, Basel, Switzerland). Individual TUNEL^+^ cells were counted in consecutive 1 mm^2^ fields in the ischemic penumbra of six randomly selected mice from each group. The number of TUNEL^+^ cells per area in 20 successive fields of view in four sections from each mouse was counted by an observer who was blinded to the study design, and the mean number of TUNEL^+^ cells in the fields of view was calculated for each mouse.

#### RNA extraction and qRT-PCR analysis

Total RNA and miRNAs were extracted from brain infarction tissues in the ischemic core by using miRNeasy Mini Kit (Qiagen, Hilden, Germany). For miR-211 analysis, cDNA was obtained using the TaqMan MicroRNA Reverse Transcription Kit (Applied Biosystems, Foster City, CA, USA) and qRT-PCR was performed using TaqMan miRNA assay kit (Applied Biosystems). U6 small nuclear RNA (U6 snRNA) was used as an endogenous control for normalization. qRT-PCR was then performed with SYBR Green Real-Time PCR Master Mixes (ThermoFisher, Waltham, MA, USA) on a 7900HT Fast RealTime PCR machine (Applied Biosystems). Changes in the miRNA levels were quantified by the 2^−ΔΔCT^ method using U6 as control. The reactions were performed in duplicate, and the number of independent experiments was marked.

#### Western blot analysis

Ischemic PC12 cells or brain tissue was homogenized by sonication in ristocetin-induced platelet aggregation (RIPA) buffer, containing protease and phosphatase inhibitors (Roche Diagnostics). Protein concentration was assessed using a Protein Assay Kit (Bio-Rad). Proteins were separated on a 12% Tris-Glycine gradient gel (Bio-Rad) from 80 to 120 V, then transferred onto nitrocellulose membranes for 1.5 h at 4°C at 80 V. Membranes were blocked in 5% BSA for 1 h at room temperature, incubated with primary antibody overnight at 4°C, washed three times for 10 min each at room temperature, incubated with an HRP-coupled secondary antibody for 1 h, and washed three times for 10 min each at room temperature. All incubations were performed in TBS buffer including 0.1% Tween-20. The following primary antibodies were used: rabbit anti-PUMA (1:1000, Cell Signaling); rabbit anti-cleaved caspase-3 (1:1000, Cell Signaling) and mouse anti-β-actin (1: 1000, Sigma-Aldrich). Finally, images of Western blots were captured by densitometry (Bio-Rad).

#### Statistical analysis

All experiments were performed at least in triplicate. Results were expressed as mean ± S.D. and analyzed by unpaired *t*-test or ANOVA in which multiple comparisons were carried out using the method of least significant difference. Differences were considered significant if the probability of the difference occurring by chance was <0.05 (*P* < 0.05).

## Results

The expression of miR-211 is decreased in PC12 subject to OGD/R and MCAO rat brain following 24 h of reperfusion

**Figure 1.**
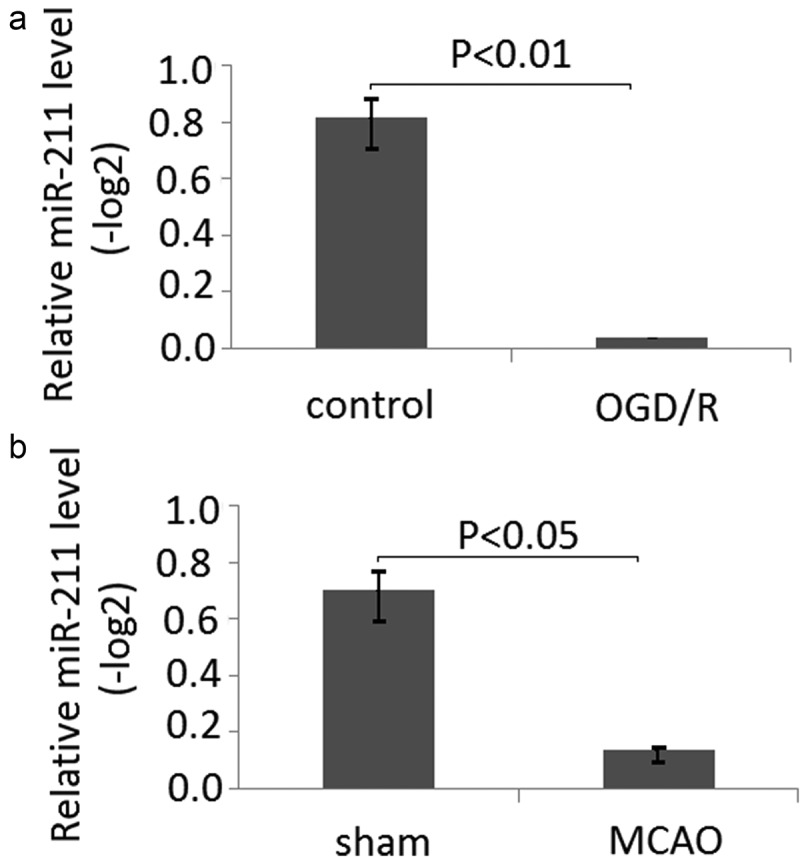
The expression of miR-211 is in PC12 subject to OGD/R and MCAO rats following 24 h of reperfusion. A, mir-211 expression was detected by RT-qPCR in PC12 subject to OGD/R; B, mir-211 expression was detected by RT-qPCR in MCAO model.

After the PC12 cells were subject to OGD/R, the relative expression of miR-211 was measured by RT-qPCR. The relative expression of miR-211 in untreated PC-12 cells was (0.81 ± 0.16)(-log2), and (0.06 ± 0.01)(-log2),which was significantly decreased compared to the untreated PC-12 cells (Figure 1(A), P < 0.01).

Following 24 h of reperfusion, all of the rats were sacrificed by CO_2_ asphyxiation. The relative expression of miR-211 was measured by RT-qPCR. Compared with the control group [(0.74 ± 0.13)(-log2)], the miR-211 expression of the MCAO group[(0.13 ± 0.08)(-log2)] was significantly decreased (Figure 1(B), P < 0.05).

Apoptosis levels are elevated in PC12 subject to OGD/R and MCAO rat brain following 24 h of reperfusion

**Figure 2.**
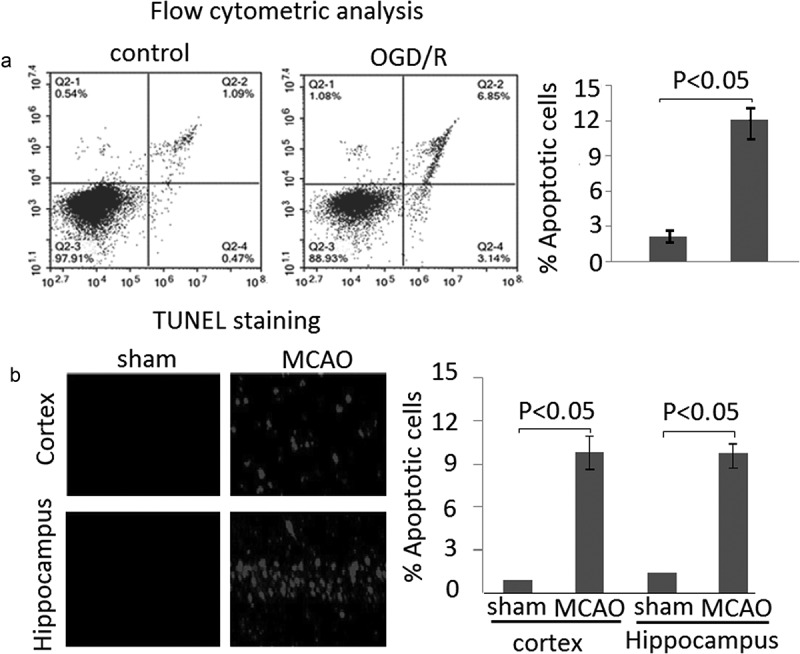
Apoptosis in PC12 cells subject to OGD/R and MCAO rats following 24 h of reperfusion. A, Apoptotic cells was measured by flow cytometric analysis. B, Apoptosis levels were detected by TUNEL staining in MCAO rats following 24 h reperfusion. Data are presented as the mean ± standard error of the mean.

Apoptosis levels were detected in PC12 cells subject to OGD/R through flow cytometric analysis. The results showed that apoptosis cells were significantly increased in PC12 cells subject to OGD/R compared to the untreated cells (Figure 2(A), P < 0.05). Rats subjected to MCAO (6 rats/group) were sacrificed at 24 h following reperfusion. The brain tissues were harvested. Apoptosis levels were detected by TUNEL staining in frozen brain sections. The results revealed that apoptosis levels was significantly increased compared to the sham. (Figure 2(B), P < 0.05)

miR-211 overexpression reduced cell apoptosis in PC12 subject to OGD/R and MCAO rat brain following 24 h of reperfusion

**Figure 3.**
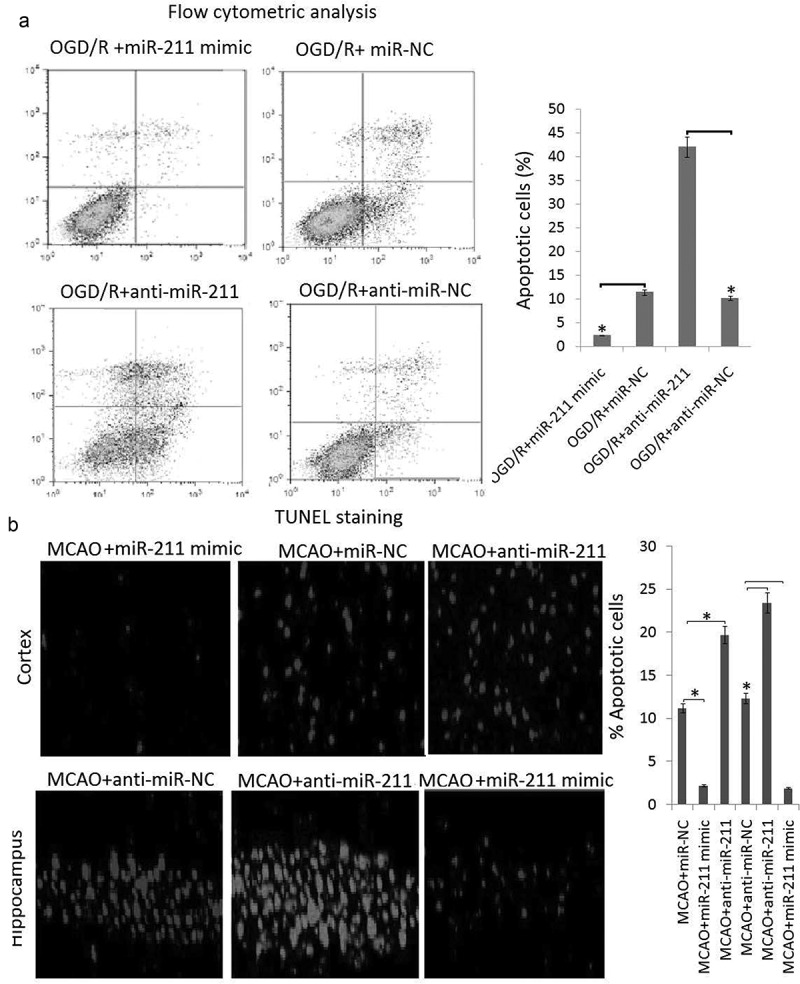
The effect of miR-211 on apoptosis in PC12 subject to OGD/R and MCAO rats following 24 h of reperfusion A, PC12 cells were transfected with miR-211 mimic or anti-miR-211 or its control, then subject to OGD/R. Apoptotic cells was measured by flow cytometric analysis. B, the rats were transfected with miR-211 mimic or anti-miR-211 or its control for 24 h, then subject to MCAO. Apoptosis levels were detected by TUNEL staining in MCAO rats following 24 h reperfusion. Data are presented as the mean ± standard error of the mean.*P < 0.05.

In order to explore the effect of miR-211 overexpression on the neuronal apoptosis, we first performed flow cytometric analysis in PC12 cells. The results showed that the apoptotic PC12 was significantly decreased in miR-211 mimic groups compared to the miR-NC groups (Figure 3(A), P < 0.05). However, the apoptotic PC12 was significantly increased in anti-miR-211 groups compared to the anti-miR-NC groups (Figure 3(A), P < 0.05).

Rats subjected to MCAO (6 rats/group) were sacrificed at 24 h following reperfusion. The brain tissues were harvested. Apoptosis levels were detected by TUNEL staining in frozen brain sections. A large number of TUNEL-positive cells was observed in the brain sections of rats subjected to MCAO, whereas TUNEL-positive cells were significantly reduced in the miR-221 mimic group compared with the miR-NC group, which was reflected by the rate of quantitated apoptosis labeled by TUNEL staining (Figure 3(B)). However, TUNEL-positive cells were significantly increased in the miR-221 inhibitor group compared with the anti-miR-NC group, which was reflected by the rate of quantitated apoptosis labeled by TUNEL staining (Figure 3(B)).

### MiR-211 mimic reduces infarction volumes in MCAO rat brain

**Figure 4.**
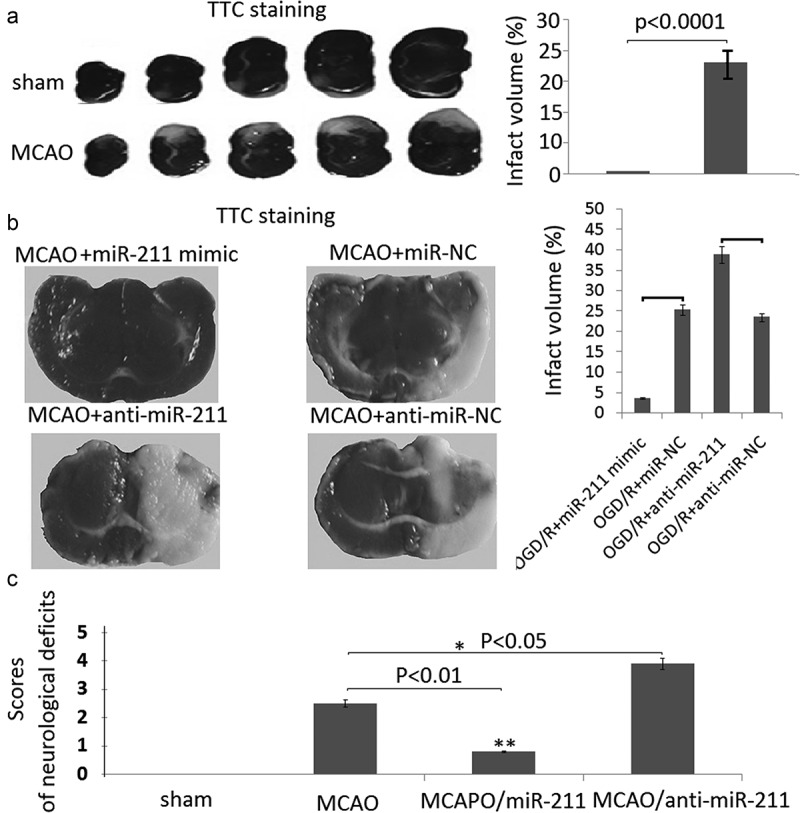
MiR-211 attenuated infarction volumes in MCAO brain. A, TTC staining of representative coronal sections after untreated MCAO. The relative infarct area percentage was evaluated by observing the unstained infarcted tissue zone (white) and the stained normal tissue zone (red). B,TTC staining of representative coronal sections after miR-211 mimic or anti-miR-211 treated MCAO. The relative infarct area percentage was evaluated by observing the unstained infarcted tissue zone (white) and the stained normal tissue zone (red). *C*, Neurological deﬁcit scores with analysis of variances followed by the Bonferroni/Dunn post hoc test).*P < 0.05;**P < 0.01.

To demonstrate the protective role of miR-211 against brain I/R injury, we first established the model of I/R rat brain and determined the effect of miR-211 mimic on infarction volumes by TTC staining. As shown in Figure 4(A), the infarct region was observed in the brain of MCAO groups. However, the infarct volume was significantly reduced in the MCAO group treated with miR-211 mimic (Figure 4(B)). The infarct volume was significantly increased in the MCAO group treated with anti-miR-211 (Figure 4(B), p < 0.01, one-way analysis of variance test).

The results of the neurological severity score was shown in Figure 4(C). There was no neurological impairment in sham rats. MCAO rats scored significantly higher on the scoring systems than rats in the other groups. Rats in the miR-211 mimic groups had lower scores than the rats in the MCAO group (*P* < 0.01). Rats in the anti-miR-211 groups had higher scores than the rats in the MCAO groups (*P* < 0.05).

### miR-211 overexpression reduced neuron apoptosis through PUMA

**Figure 5.**
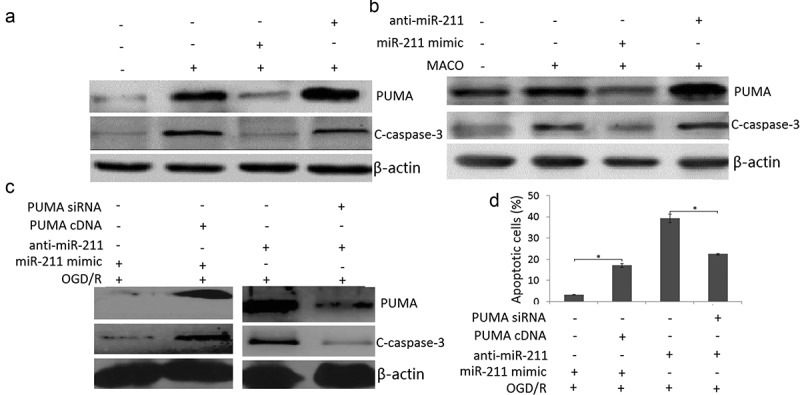
PUMA inhibition by miR-211. A, Western blot assay was used to detect PUMA and cleaved-caspase-3 expression in OGD/R or/and miR-211 mimic or OGD/R or/and anti-miR-211 treated PC12 cells. B, Western blot assay was used to detect PUMA and cleaved-caspase-3 expression in MCAO + miR-211 mimic or MCAO + anti-miR-211 treated rat brain. C, Western blot assay was used to detect PUMA and cleaved-caspase-3 expression in (OGD/R + miR-211 mimic+ PUMA cDNA) or (OGD/R + anti-miR-211 mimic+ PUMA siRNA) treated PC12 cells. D, PC12 cells were transfected with miR-211 mimic + PUMA cDNA or anti-miR-211+ PUMA siRNA, then subject to OGD/R. Apoptotic cells was measured by flow cytometric analysis.*P < 0.05.

To determine whether miR-211 regulate the apoptosis of PC12 cells through PUMA, we detected PUMA expression by western blot assay in OGD/R or/and miR-211 mimic or anti-miR-211 treated PC12 cells. The results showed that OGD/R or MCAO upregulated PUMA expression and activated caspase-3 expression in the PC12 cells (Figure 5(A)) or MCAO rat brain (Figure 5(B)). However, miR-211 mimic abolished the promoter effect, and anti-miR-211 enhanced the promoter effect in the PC12 cells (Figure 5(A)) or MCAO rat brain (Figure 5(B)). The control miR-NC and anti-miR-NC has not effect on PUMA expression and activated caspase-3 expression in the PC12 cells or MCAO rat brain (data not shown).

We then co-transfected pcDNA.3-PUMA and miR-211 mimic into PC12 cells 24 h prior to OGD/R treatment. The results showed that PUMA expression was restored in miR-211 mimic transfected PC12 cells (Figure 5(C)); We next co-transfected PUMA siRNA and anti-miR-211 into PC12 cells 24 h prior to OGD/R treatment. The results showed that PUMA expression was reversed in anti-miR-211 transfected PC12 cells (Figure 5(C)); Furthermore, PUMA overexpression abolished the inhibition effect of miR-211 mimic on OGD/R-induced apoptosis in PC12 cells (Figure 5(D)); Targeting PUMA abolished the promotor effect of anti-miR-211 on OGD/R-induced apoptosis in PC12 cells (Figure 5(D)). These findings indicated that miR-211 overexpression reduced PC12 cell apoptosis through PUMA downregulation.

## Discussion

Numerous studies have demonstrated that expressions of miRNAs would be changed in the brain in response to I/R [24,35,36]. Elucidation of specific miRNA is thus considered as a potential target against I/R injury. In the present study, we demonstrated that miR-211 expression is significantly down-regulated in the rat brain with MCAO. With the *in vivo* and *in vitro* MCAO models, we found that miR-211 mimic provides significant protection from injury of PC12 cells subject to OGD/R and rats subject to MCAO, which is reflected by reduced infarct volumes and cell apoptosis. Mechanistically, our findings showed that miR-211-mediated protective effects are associated with PUMA downregulation.

miR-211 is located on intron 6 of the Trpm1 gene at 15q13-q14, a locus that is frequently lost in many neoplasms [37–39]. Accumulating evidences have demonstrated that miR-211 expression was down-regulated in rat model of hypoxic-ischemic injury, ganglion cell dysplasia in congenital intestinal atresia and Epilepsy [40–42].

However, it is not clear whether miR-211 l was associated with the brain injury induced by I/R.

Rat pheochromocytoma line 12 (PC12) cell provides a useful model system for neurological and neurochemical studies [43]. In PC12 cell, neuronal apoptosis may be due to different apoptotic pathways: intrinsic or extrinsic for example [44]. Previous study reported that PC12 cells from OGD/R injury partially by the JAK2/STAT3-dependent inhibition of apoptosis, which provided a novel therapeutic target for the treatment of cerebral I/R injury [45].

In the present study, miR-211 was downregulated in OGD/R-treated PC12 cells, and its downregulation promoted OGD/R treatment-induced PC12 injury, as revealed by increased cell apoptosis. However, enforced miR-211 relieved OGD/R treatment-induced PC12 injury, as revealed by decreased cell apoptosis. p53 upregulated modulator of apoptosis (PUMA) binds and antagonizes all known antiapoptotic Bcl2 family members and activates two key multidomain proapoptotic Bcl2 family proteins, BAX and BAK, leading to mitochondrial dysfunction and caspase activation [46]. Li et al. [47] reported that postconditioning can reduce I/R-induced cardiomyocyte apoptosis by targeting PUMA, which is involved in lethal I/R injury. In the study, it was observed that PUMA was the target of miR-211, and that the effects of enforced miR-211 on OGD/R-treated PC12 cells were eliminated by enforced PUMA expression, and miR-211 inhibition on OGD/R-treated PC12 cells were eliminated by PUMA silencing. The data suggest that enforced miR-211 reduced PUMA -dependent cell apoptosis in OGD/R treated PC12 cells.

To further clarify the protective effect of miR-211 in vivo, the MCAO model was established. 24 h after MCAO model establishment, the expression of miR-211 in the cerebral cortex on the ischemic side was found to be significantly lower than the sham groups. Correspondingly, PUMA and cleaved-caspase-3 protein levels were significantly high than the sham groups. PUMA has reported to be invovled in the apoptosis of cerebral astrocytes upon I/R injury, and targeting PUMA could reverse the effect [18]. In the present study, enforced miR-211 in the MCAO model had comparably smaller cerebral infarct size and reduced neuronal apoptosis followed by decreased PUMA and cleaved-caspase-3 expression. However, miR-211 inhibition in the MCAO model had comparably larger cerebral infarct size and increased neuronal apoptosis followed by increased PUMA and cleaved-caspase-3 expression. However, whether miR-211 can directly block PUMA upregulation and activate caspase-3 in vivo concurrently needs to be further investigated.

### Conclusion

Our findings demonstrated that miR-211 was downregulated in the ODG/R treated PC12 cells and MCAO treated rat model. Enforced miR-211 significantly reduced cell apoptosis in ODG/R or MCAO treated PC12 cells or rat model, and vice versa. Furthermore, we also demonstrated that miR-211 enhanced the protective effects to ODG/R or MCAO induced cell or brain injury by targeting PUMA. Therefore,miR-211-PUMA interaction will aid the design of new strategies for the therapeutic interventions in cerebral ischemic stroke.

## Highlight

1. miR-211 was downregulated in OGD/R or MCAO treated PC12 cells or rat brain.
2. Enforced miR-211 inhibited cell apoptosis on OGD/R or MCAO treated PC12 cells or rat brain.
3. Targeting miR-211 promoted cell apoptosis on OGD/R or MCAO treated PC12 cells or rat brain.
4. Enforced miR-211 reduced infarct volumes and neurologic score in MCAO treated rat brain.
5. miR-211-mediated protective effects are associated with targeting PUMA in *in vitro* and *in vivo*.
